# Unlocking the Biocontrol Potential of Indigenous Soil Fungi: High-Performing Strains of *Beauveria bassiana* and *Metarhizium robertsii* Against the Tomato Leafminer *Tuta absoluta*

**DOI:** 10.3390/jof12060452

**Published:** 2026-06-21

**Authors:** Noureddine Idali, Abdelhi Dihazi, Mohammed Lahcini, Tariq Butt, Abdellatif El Meziane

**Affiliations:** 1 Laboratory of Excellence in Agrobiotechnology and Bioengineering, AgroBioTech Center, CNRST-Labeled Research Unit (URL05-CNRST), Cadi Ayyad University, Marrakech 40000, Morocco; 2 IMED-Lab, Cadi Ayyad University (UCA), Avenue Abdelkrim Elkhattabi, B.P. 549, Marrakech 40000, Morocco; 3 High Throughput Multidisciplinary Research Laboratory (HTMR), Mohammed VI Polytechnic University (UM6P), Ben Guerir 43150, Morocco; 4 Department of Biosciences, Swansea University, Singleton Park, Swansea SA2 PP, UK

**Keywords:** entomopathogenic fungi, EPF collection, multi-trait screening, biological control, *Tuta absoluta*, *Beauveria bassiana*, *Metarhizium robertsii*

## Abstract

The invasive tomato leafminer, *Tuta absoluta*, poses a severe global threat to solanaceous crops, necessitating sustainable biocontrol solutions. Through systematic bioprospecting across several Moroccan soils, we constructed a novel library of indigenous fungal isolates using complementary *Tenebrio molitor* baiting and selective media methods. High-throughput phenotyping identified 49 highly pathogenic isolates, which were characterized for conidial production, thermotolerance, and virulence against *T. absoluta*. We discovered two lead isolates, *Beauveria bassiana* UCA-350 and *Metarhizium robertsii* UCA-329, that demonstrated superior virulence, reducing median survival time and achieving lower LC_50_ values than most commercial reference strains. Notably, virulence was positively correlated with in vitro conidial yield, revealing a key trait linkage for strain selection. Furthermore, genus-level divergence in thermotolerance was observed, with *Beauveria* isolates exhibiting significantly higher heat resilience. Our integrated multi-trait screening pipeline not only delivers two potent, regionally sourced biocontrol candidates but also establishes a phenotypic selection framework that prioritizes both efficacy and production scalability, advancing the rational development of next-generation mycoinsecticides.

## 1. Introduction

The global agriculture landscape faces a dual crisis: the urgent need to ensure food security for a growing population and the imperative to mitigate the profound environmental and societal costs of intensive pesticide use. The development of insecticide resistance in over 600 arthropod species, coupled with the destabilization of ecosystem services and risks to human health, has rendered the status quo of chemical-dependent pest management untenable [1,2]. This confluence of challenges has been the impetus for a paradigm shift toward Integrated Pest Management (IPM), wherein biological control agents form a critical pillar. Among these agents, entomopathogenic fungi (EPF), particularly hypocrealean ascomycetes EPF, have gained significant and sustained scientific interest due to their broad host range and self-dissemination mechanisms, offering a promising route to sustainable pest suppression [3,4,5].

Hypocrealean EPF, particularly the *Beauveria*, *Metarhizium*, *Isaria*, and *Lecanicillium* genera, are facultative pathogens that contribute to the natural regulation of arthropod populations across soil and phyllosphere habitats [6,7]. The infection process begins with the adhesion of hydrophobic conidia to the insect integument, followed by germination and the formation of appressoria [8]. Through a combination of immense turgor pressure and the secretion of a cocktail of cuticle-degrading enzymes, the fungus breaches the host’s physical barrier [8,9,10,11]. Once within the hemocoel, the fungus undergoes a dimorphic shift to yeast-like hyphal bodies (blastospores), proliferating while actively modulating and suppressing the host immune system. Host death results from a combination of nutrient depletion, tissue destruction, colonization, and the action of fungal toxins. Critically, under conditions of high humidity, the cadaver becomes a site of prolific external conidiation, releasing millions of new infectious propagules into the environment to initiate secondary infection cycles—a feature that underpins their potential for causing epizootics and ensuring environmental persistence [8,12,13,14].

Biocontrol strategies often relied on a handful of commercialized, generalist EPF strains deployed across diverse geographies [15]. However, a growing body of evidence reveals that this “one-size-fits-all” approach may be suboptimal. Fungal performance is intimately tied to environmental adaptation. Traits with thermotolerance and compatibility with local microbial communities are finely tuned by evolution. Consequently, the global trend has decisively shifted towards the prospection, characterization, and utilization of indigenous EPF strains, which are naturally selected for and pre-adapted to local climatic conditions and agroecosystems [16,17,18,19].

This local adaptation paradigm is consistently validated by comparative studies. For instance, indigenous *B. bassiana* strains from Puerto Rico matched the efficacy of the commercial *B. bassiana* GHA against the coffee berry borer in the laboratory, but more importantly, their pathogenicity and persistence exceeded in the field [20]. Similarly, locally isolated *B. bassiana* strains from spotted lanternfly populations in the eastern USA outperformed the commercial *B. bassiana* strain GHA against *Lycorma delicatula* adults and nymphs [21]. Furthermore, studies on whiteflies (*Bemisia tabaci* and *Trialeurodes vaporariorum*) and African cotton leafworm (*Spodoptera littoralis*) have repeatedly found native isolates with superior pathogenicity to common commercialized strains like *B. bassiana* ATCC 74040 [22,23]. This empirical justification makes the construction and rigorous evaluation of localized EPF libraries a critical first step in developing resilient, context-specific biocontrol solutions.

A compelling example of the urgent need for innovative pest management strategies is the tomato leafminer, *Tuta absoluta* (Meyrick) (Lepidoptera: Gelechiidae). Native to South America, this pest has become a global threat to tomato (*Solanum lycopersicum*) production, capable of causing yield losses of 80–100% in the absence of effective control [24,25]. Its invasion of the Mediterranean basin, reaching Morocco around 2008, has been particularly destructive, facilitated by favorable climatic conditions and the widespread practice of year-round tomato cultivation [26].

The biology of *T. absoluta* poses exceptional challenges to control. Its larval leaf-mining behavior provides substantial protection from contact insecticides. Its high fecundity and short generation time are further accelerated by elevated temperatures that often suppress the performance of many biocontrol agents (BCAs), and it has rapidly evolved resistance to multiple insecticide classes [27,28,29]. This convergence of concealment, rapid population growth, and resistance underscores the need for alternative control agents capable of reaching cryptic larvae, acting rapidly, and maintaining efficacy under harsh field conditions.

Accordingly, the evaluation of EPF for *T. absoluta* management cannot rely solely on laboratory virulence assays, which can be poor predictors of field performance. Instead, a holistic, multi-trait selection framework is required. First, high in vitro conidial yield and vigor are essential prerequisites for cost-effective mass production and for achieving sufficient propagule pressure to offset environmental losses and ensure infection [30,31]. Robust sporulation can accelerate host mortality, while prolific conidiation on insect cadavers—especially the production of abundant secondary inoculum—enhances epizootic potential and population-level suppression [32,33,34]. Second, thermotolerance is a critical determinant of persistence and efficacy in open-field tomato systems, where canopy temperatures might exceed 40 °C. Conidial viability, germination kinetics, and pathogenic activity are all highly susceptible to heat stress, making systematic screening for thermal resilience indispensable when targeting warm agroecosystems [31,35,36].

To address these challenges, we conducted a comprehensive study aimed at discovering and characterizing indigenous EPF from Moroccan soils, with particular emphasis on the endemic, arid-adapted *Argania spinosa* forest ecosystem. The study was structured around three sequential and interlinked objectives: (i) establish a diverse EPF collection using a dual-isolation strategy—combining *Tenebrio molitor* baiting with selective media cultivation—to mitigate the taxonomic and functional biases inherent in single-method approaches; (ii) morphologically characterize recovered isolates to enable accurate genus-level identification; and (iii) implement a multi-trait screening pipeline that evaluated not only pathogenicity against *T. absoluta* but also two critical enabling traits: conidial production capacity and thermotolerance. Through this integrated approach, we sought to move beyond the identification of merely virulent isolates and instead prioritize robust, high-performing, and locally adapted EPF candidates possessing the trait combinations necessary for successful development into effective mycoinsecticides against *T. absoluta* and other recalcitrant pests.

## 2. Materials and Methods

### 2.1. Soil Sampling

Soil samples were collected between October 2022 and March 2023 from different regions in Morocco, including cultivated lands and forest areas (Table S1). The samples were taken from the top 5–15 cm of the soil, where fungal spores are most abundant [37]. A total of 70 samples were collected, and each sample was stored in sterile plastic bags, labeled with precise location data, and stored at 4 °C until required.

### 2.2. Fungal Isolation

To isolate EPF from the collected soil samples, two complementary methods were used: The first one involved the *Tenebrio molitor*-baiting technique, in which the larvae of *T. molitor* (4th–5th instar) were used as bait to attract fungal pathogens. following the same protocol of Kim et al. (2018) [17].

In the second method, a selective medium was used to directly isolate fungal propagules from the soil, as shown in ref. [37] with changes. Briefly, thirty grams of soil from each sample was suspended in 250 mL of sterile 0.05% Triton-X solution, and the suspension was shaken for one hour. One ml of the resulting suspension was spread onto quartered Sabouraud Dextrose Agar medium (¼SDA) supplemented with cycloheximide (0.02%) and chloramphenicol (0.05%). Once the fungal colonies were observed, individual colonies were subcultured and purified and then transferred onto potato dextrose agar supplemented with yeast extract (PDAY) to facilitate the development of pure fungal cultures. The cultures of purified isolates were stored in either sterilized distilled water or mineral oil, following the protocol described by Ayala-Zermeño et al. [38].

#### Preliminary Pathogenicity Screening Using *T. molitor*

The pathogenic potential of the isolates was preliminarily assessed using Tenebrio molitor larvae. Each isolate was cultured on ¼SDA at 26 °C for 10 days. Ten healthy fourth-instar larvae were placed on each fungal culture, with three replicate plates per isolate [16,17]. Control larvae were placed on fungus-free ¼SDA plates. All plates were incubated in darkness at 26 °C for 10 days without supplementary diet, and the experiment was repeated three times. Mortality was recorded daily over 10 days. Dead insects were surface sterilized by sequential immersion in 70% ethanol for 30 s, followed by 1% (*v*/*v*) sodium hypochlorite for 30 s, then rinsed three times in sterile distilled water (1 min each). The cadavers were then transferred to petri dishes lined with moistened tissue paper to encourage external sporulation. Isolates causing larval mortality within 10 days were retained for further testing.

### 2.3. EPF Conidial Viability

The conidial viability was evaluated by determining the ability of conidia to germinate, following [39]. The conidial suspension was prepared and adjusted to 10^7^ conidia/mL for each isolate. A 10 µL aliquot of each suspension was evenly spread onto ¼SDA plates and incubated for 16 h at 26 °C in the dark. The ability to germinate was examined using a light microscope, and the conidium was considered germinated when its germ tube reached the conidium’s diameter. At least 300 conidia were counted for each isolate, and the viability was expressed as the percentage of germinated conidia. Only isolates exhibiting more than 80% as conidial viability were kept for further experiments.

### 2.4. Conidia Production by EPF

Conidial production was quantified following Kim et al. [17] with minor modifications. Reference benchmark strains included *Beauveria bassiana* ATCC 74040, *B. bassiana* GHA (=ARSEF 6444), *Metarhizium brunneum* V275 (=Met52/BIPESCO 5), and *M. brunneum* ARSEF 4556. Isolates ATCC 74040, GHA and V275 are the active ingredients in Naturalis^®^ (CBC Europe Srl, Grassobbio, Italy), BotaniGard^®^ (Certis Biologicals, Columbia, MD, USA) and Lalguard M52 OD^®^ (Lallemand Inc., Montreal, QC, Canada), respectively.

Conidia were harvested from actively growing cultures on ¼SDA, adjusted to 1 × 10^7^ conidia mL^−1^, and 10 µL aliquots were spread onto agar plates. Plates were incubated in darkness at 26 °C for 10 days. Conidia were recovered from the entire agar surface with sterile 0.05% Triton X-100, then vortexed at 3000 rpm for 5 min, and sonicated to disperse aggregates. Conidial concentration was determined by using a hemocytometer, and total conidia per plate were calculated. Experiments were performed in three biological replicates and repeated three times.

### 2.5. Thermotolerance

Thermotolerance was evaluated using conidial suspensions (1 × 10^7^ conidia mL^−1^) prepared in triplicate for each isolate, following Rangel et al. [40]. One-milliliter aliquots were placed in sterile microcentrifuge tubes and incubated at 45 °C for 2 h, while control suspensions were maintained under identical conditions without heat treatment. After incubation, 10 µL of each suspension was plated onto ¼SDA and incubated at 26 °C for 16 h. Germination was scored as a conidium with a germ tube length equal to or greater than the conidial diameter. Germination rate was determined by scoring at least 300 randomly selected conidia per sample, and isolates were classified as thermotolerant when relative germination was ≥60%.

### 2.6. Tuta absoluta Rearing

Tomato leaves and fruits infested with *T. absoluta* larvae and pupae were collected from fields in the Souss Massa Daraa region and reared in BugDorm cages in the greenhouse. Fresh tomato leaves were supplied weekly for oviposition and larval feeding. The infested plant material was subsequently transferred to 3–5-week-old potted tomato plants (cv. ‘Campbell 33’).

### 2.7. EPF Virulence Against Tuta absoluta

The pathogenicity of fungal isolates was assessed at 1 × 10^8^ conidia/mL against third–fourth instar *T. absoluta* larvae using a modified IRAC leaf-dip bioassay [41]. Tomato leaves were dipped in conidial suspension for 10 s, then the petioles were wrapped with sterile, moistened cotton and placed in Petri dishes. Ten larvae were added per dish, with three replicates per isolate (30 larvae total). Control leaves were treated with sterile 0.05% Triton X-100. All treatments were maintained under controlled conditions for 10 days (26 ± 2 °C, 60% RH, in total darkness), and larval mortality was recorded daily. Virulence was estimated using median survival time (MST), with lower values indicating higher pathogenicity. Isolates with log(HR) ≥ 2 relative to *B. bassiana* ATCC 74040 were selected for dose–response assays.

### 2.8. LC_50_ and LC_90_ Determination for the Most-Performing Isolates

To further characterize the most virulent isolates, the lethal concentration (LC_50_) values were determined using three conidial suspension concentrations: 1 × 10^5^, 1 × 10^6^, and 1 × 10^7^ conidia/mL. The same leaf-dip method was employed for each concentration, and the experiment was conducted in triplicate, with 10 larvae per replicate. The leaves treated with sterile Triton-X solution served as a control. Larval mortality was recorded daily over 10 days. Bioassays for each concentration were performed under the same controlled conditions described above.

### 2.9. Morphological and Molecular Identification of the EPF

The morphological identification of the most performing isolates was carried out over a period of 7 to 14 days by determining macroscopic characteristics of the fungal colonies and by optical microscope after staining with lactophenol cotton blue [42].

The molecular identification was conducted using a multi-locus approach targeting three genomic regions: the internal transcribed spacer (ITS), the elongation factor 1-alpha (EF1-α), and the nuclear block region (Idali et al. paper in preparation on the diversity of the Moroccan EPF).

### 2.10. Statistical Analysis

All analyses and graphics were performed in R version 4.5.1 [43], with a significance level of α = 0.05. Conidial counts per plate were log_10_-transformed and analyzed by one-way ANOVA, with Tukey’s HSD for pairwise comparisons and a Welch two-sample *t*-test to compare genera. Thermotolerance (germination after heat exposure vs. control) was assessed using Fisher’s exact tests with Benjamini–Hochberg FDR correction, and genus differences under heat treatment were evaluated with a quasibinomial generalized linear model. Pathogenicity and dose-response survival data were analyzed using Kaplan–Meier curves and log-rank tests, and Cox proportional hazards models were fitted to estimate hazard ratios and median survival times. LC_50_ and LC_90_ values for the selected isolates were obtained from two-parameter log-logistic models after Abbott correction for control mortality. Relationships between conidia production, thermotolerance, and virulence (log hazard ratio) were explored using Pearson correlations visualized with a principal component analysis (PCA) biplot based on standardized variables.

## 3. Results

### 3.1. Construction and Primary Screening of a Novel Entomopathogenic Fungal Library

**Figure 1:**
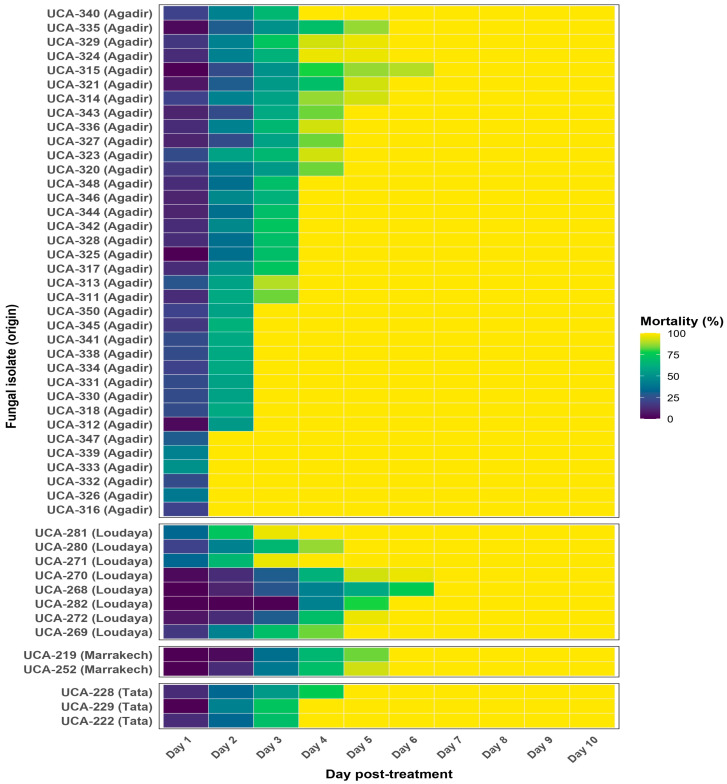
Heatmap of the pathogenicity screening of EPF against *T. molitor*. The daily mortality (%) of *T. molitor* larvae was followed over 10 days for each fungal isolate. Mortality was recorded for each isolate from dark blue (0%) to yellow (100%).

To establish a regionally sourced library of EPF, soil samples from four distinct Moroccan biotopes were subjected to two complementary isolation techniques: *Tenebrio molitor* baiting (TBM) and selective medium cultivation (SMC). This dual approach yielded a collection of 144 distinct fungal isolates, 88 with the selective medium (41 from Marrakech, 24 from Loudaya, 7 from Agadir and 16 from Tata) and 56 with the baiting method (8 from Marrakech, 14 from Loudaya and 34 from Agadir) (Figure S1, Table S1). The first pathogenicity screening of these 144 fungal isolates on *T. molitor* showed that a total of 49 isolates of the collection caused 100% larval mortality within 10 days (Figure 1, Table S2). Among these EPF isolates, 38 were isolated by TBM, and only 11 were isolated by SMC. This step reduced the initial collection to 49 most promising strains forming the core of our EPF library.

### 3.2. Morphological Identification of EPF Library

The morphological examination of the EPF isolates was carried out using macroscopic and microscopic observations and revealed two main genera: *Beauveria* and *Metarhizium* (Figure S2). *Beauveria* isolates typically formed white to cream, powdery colonies, whereas *Metarhizium* isolates formed greenish colonies that darkened with age. Microscopic features were also consistent with these genera.

### 3.3. Conidia Production Assay

**Figure 2:**
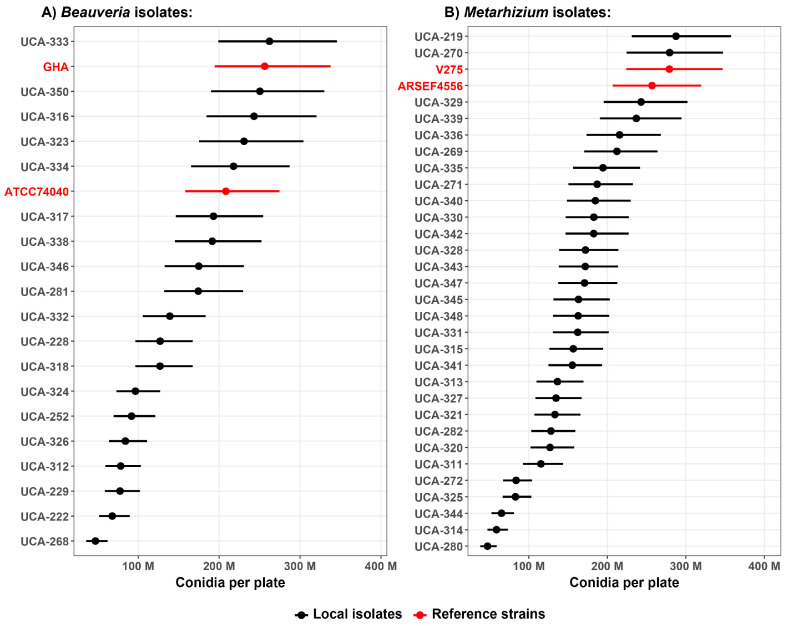
Mean conidial production of *Beauveria* (**A**) and *Metarhizium* (**B**) isolates after 10 days incubation on ¼SDA medium ± 95% CI. The reference strains (*B. bassiana* ATCC 74040 and GHA (ARSEF 6444); *M. brunneum* F52, V275, ARSEF 4556) were highlighted in red.

The conidial yield of the 49 isolates varied markedly after 10 days of incubation on SDA medium, and the *Beauveria* isolates produced between 26.22 × 10^7^ (UCA-333) and 4.72 × 10^7^ conidia (UCA-268), whereas the *Metarhizium* ones yielded 28.74 × 10^7^ (UCA-219) and 4.74 × 10^7^ (UCA-280) (Figure 2, Table S3). The highest conidial yields were obtained by nine *Beauveria* and six *Metarhizium* isolates. These yields are not significantly different from those obtained from the marketed strains *B. bassiana* GHA and ATCC 74040 and *M. brunneum* V275, and ARSEF 4556 (Table S4). These results showed that several isolates from our library performed equally to the reference strains.

### 3.4. Heat Tolerance Assay

The thermal stress experiments showed that the germination rates of the *Beauveria* isolates UCA-222 and UCA-252, and the *Metarhizium* isolates UCA-269 and UCA-282, were not significantly different after exposure to 45 °C for 2 h (Table S5). These 4 isolates were highly tolerant to thermal stress. Furthermore, an additional eight *Beauveria* isolates maintained more than 50% of their initial germination rate and are considered moderate in their tolerance to thermal stress.

**Figure 3:**
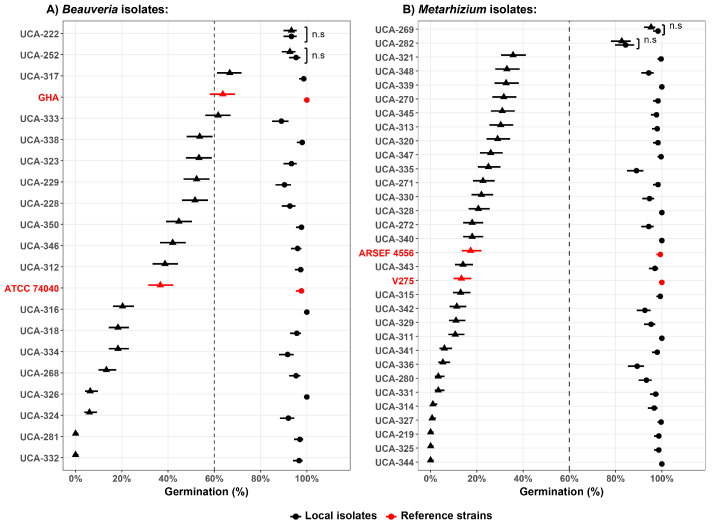
Thermotolerance of *Beauveria* (**A**) and *Metarhizium* (**B**) isolates after heat treatment (2 h at 45 °C). The thermotolerance was evaluated by the percentage of the conidial germination (%) after heat treatment (● control and ▲ heat-treated). Points are means with Wilson 95% Cis. Red indicates the reference strains. The vertical dashed line marks the 60% thermotolerance threshold. “n.s” indicates no significant difference between treatment and control (Fisher’s exact test with BH FDR correction, q ≥ 0.05).

The heat exposure revealed a significant difference in germination between the *Beauveria* and *Metarhizium* genera from our entomopathogenic fungal library (Figure 3). *Beauveria* maintained an overall higher mean germination than *Metarhizium* under heat stress (38.6%, 95% CI 27.3–51.3% vs. 21.1%, 95% CI 14.1–30.4%; *p* = 0.019). In this study, the reference strain *B. bassiana* GHA maintained more than 50% of the germination rate (64%), whereas all the other reference strains were strongly affected by the heat treatment: V275 (13%) and ARSEF 4556 (17%).

### 3.5. Pathogenicity Trials Against T. absoluta

**Figure 4:**
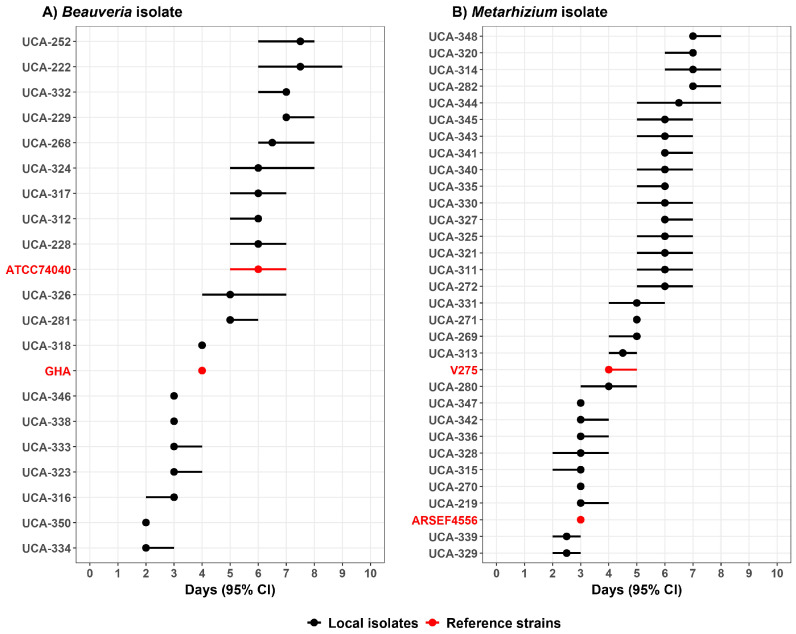
Median survival times in days (MST) of *T. absoluta* larvae following infection by the entomopathogenic fungal isolates: (**A**) *B. bassiana* and (**B**) *Metarhizium*. The points show the MST from Kaplan–Meier estimates, and horizontal bars are 95% CIs. The reference strains were presented in red.

The results showed that the larval survival of *T. absoluta* varied strongly depending on the isolates (log-rank; *p* < 0.001), but not between the two genera (log-rank; *p* = 0.3). The median survival times (MSTs) ranged from 2 to 9 days across the collection (Figure 4), indicating that variation is primarily driven by isolates rather than genus. To identify the isolates exerting the strongest impact on *T. absoluta* larvae, we fitted a Cox proportional hazards model using *B. bassiana* ATCC 74040 (MST = 6 days) as the baseline and selected isolates with a log hazard ratio (log(HR)) ≥ 2 relative to this strain (Table S6). Within *Beauveria*, seven isolates met this criterion: UCA-350 (MST = 2 days), UCA-334 (MST = 2 days), UCA-316, UCA-346, UCA-338, UCA-323, and UCA-333 (all MST = 3 days). Within *Metarhizium*, nine isolates met the same criterion: UCA-329 (MST = 2.5 days), and UCA-339, UCA-315, UCA-328, UCA-336, UCA-342, UCA-347, UCA-219, and UCA-270 (all MST = 3 days). The four additional reference strains had MSTs between 3 and 4 days. For further screening of the best pathogenic isolates to *T. absoluta,* seven *Beauveria* isolates and nine *Metarhizium* isolates were further analyzed in dose-response assays.

### 3.6. Dose-Response and Survival Patterns for the Most Virulent Isolates

**Figure 5:**
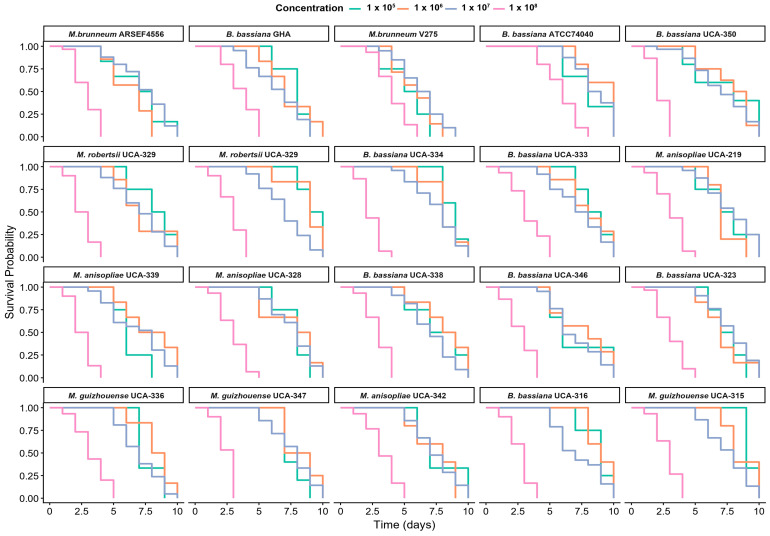
Kaplan–Meier survival curves illustrating time-to-death patterns in *T. absoluta* larvae for the 16 high-hazard log(HR) ≥ 2 and the reference strains (*B. bassiana* ATCC 74040, GHA (ARSEF 6444); *M. brunneum* F52, V275, ARSEF 4556) in the concentration 1 × 10^5^, 1 × 10^6^, 1 × 10^7^, and 1 × 10^8^ conidia/mL. Curves show survival probability over 10 days.

Across the best selected isolates, the concentrations for 50% (LC_50_) values ranged from 1.49 × 10^6^ to 7.54 × 10^6^ conidia/mL, whereas the LC_50_ for the reference strains spanned from 2.25 × 10^6^ conidia/mL for *M. brunneum* ARSEF 4556 to 1.42 × 10^7^ conidia/mL for *B. bassiana* ATCC 74040 (Table 1). The lowest LC_50_ were observed for *B. bassiana* UCA-350 (1.49 × 10^6^), *M. robertsii* UCA-329 (2.67 × 10^6^), and a cluster having an LC_50_ close to 2.91 × 10^6^ and presented by *B. bassiana* UCA-334, *B. bassiana* UCA-333, and *M. anisopliae* UCA-270. In contrast, isolates such as *M. guizhouense* UCA-315 and *B. bassiana* UCA-316 had the highest LC_50_ values with 7.54 × 10^6^ and 5.24 × 10^6^, respectively. Within the reference strains, *M. brunneum* ARSEF 4556 sat among the more potent isolates (2.25 × 10^6^), and *B. bassiana* GHA, *M. brunneum* V275 was intermediate (4.07 × 10^6^–4.43 × 10^6^), and *B. bassiana* ATCC 74040 had the highest LC_50_ value (14.2 × 10^6^). These patterns were largely mirrored by the LC_90_ values, *B. bassiana* UCA-350 and *M. robertsii* UCA-329 having the lowest LC_90_ values (7.73 × 10^6^ and 1.95 × 10^7^, respectively)_,_ and the highest LC_90_ registered by UCA-315 (7.18 × 10^7^), and *B. bassiana* UCA-316 (4.76 × 10^7^). Similarly, the reference strains ranked the same trend, with *M. brunneum* ASREF 4556 having the lowest LC_90_ (2.25 × 10^7^), followed by *B. bassiana* GHA (3.67 × 10^7^), *M. brunneum* V275 (4.29 × 10^7^), and with *B. bassiana* ATCC 74040 having the highest LC_90_ (1.13 × 10^8^). Taken together, the dose-response analysis identified *B. bassiana* UCA-350 and *M. robertsii* UCA-329 as the most potent isolates exceeding reference strains. Kaplan–Meier survival profiles across concentrations (Figure 5) mirrored this ordering, showing the fastest declines for these isolates in comparison to the reference strains.

These highly pathogenic isolates of *T. absoluta* were identified using the molecular multi-locus approach: ITS, nuclear block region, elongation factor EF1-a (Idali et al., paper in preparation). The isolates UCA-350 and UCA-329 were identified as *B. bassiana* and *M. robertsii*, respectively.

### 3.7. Inter-Trait Correlations Linking Conidia Production, Thermotolerance, and Virulence

**Figure 6:**
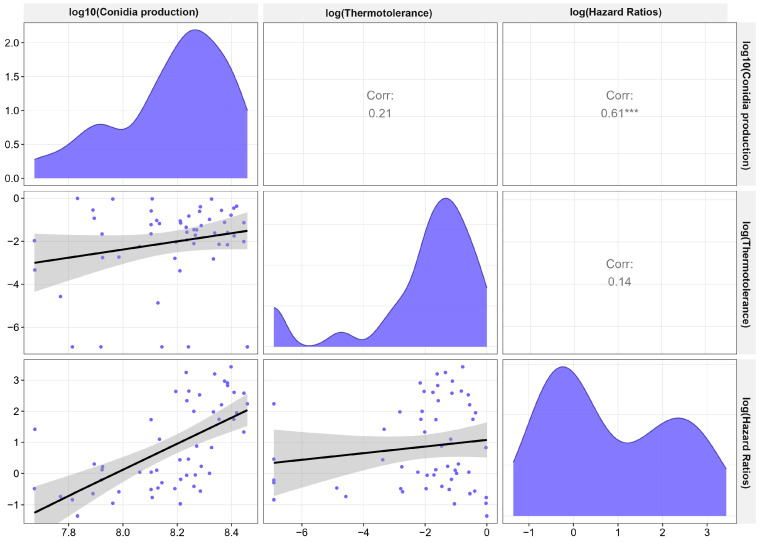
Pairs plot of log10 conidia production, log thermotolerance, and log Hazard Ratios. Upper panels: Pearson’s r, lower: Scatter with linear fit (±95% CI), and diagonal: kernel densities. Asterisks (***) indicate the significance levels of the correlations (*p* < 0.001).

Pairwise Pearson correlations showed that virulence (log(HR)) was positively associated with conidia production (*r* = 0.61), while associations were weak between production and thermotolerance (*r* = 0.21) and between thermotolerance and virulence (*r* = 0.14) (Figure 6, Table S7). A PCA of standardized traits yielded PC1 (56.7%) aligned with production and virulence and PC2 (30.5%) driven mainly by thermotolerance, mirroring the correlations (Figure S4). Descriptively, the best performing strains cluster on negative PC1 (conidia production-virulence direction), the other isolates are more dispersed toward positive PC1, and reference strains lie near the biplot origin.

## 4. Discussion

In this study, we established an entomopathogenic fungal library with a dual use of *T. molitor* baiting and selective medium cultivation. Following a preliminary pathogenicity screen against *T. molitor* larvae, 49 isolates induced significant mortality within 10 days of incubation and were then classified as putative EPF (Figure 1, Table S2). The baiting method resulted in the screening of more isolates than the selective medium, confirming the efficiency of insect-bait methods in EPF library construction [17,44]. Our collection includes *Beauveria* and *Metarhizium* spp. predominantly (Figure S2), confirming earlier findings reporting that these genera frequently occur in both agricultural and forest soils [37,45].

The combination of these complementary isolation techniques is important for the development of a diverse EPF library, as each method can bias the recovery of particular fungal taxa [46]. A selective isolation medium can promote the culture of certain fungal strains that might be highly specific to an insect species, and not pathogenic to the *Tenebrio*- or *Galleria*-baiting method alone. Masoudi et al. [47] observed that, when focusing on *Metarhizium* isolates, some variations in entomopathogenic potential have been missed using *T. molitor* solely. Therefore, a dual strategy, combining both a selective medium and an insect-bait approach, is recommended to ensure the collection of a broader range of EPF, maximizing the likelihood of obtaining isolates with strong biocontrol potential.

Geographic origin can influence the pool of recoverable EPF, and we noted that several highly virulent isolates were recovered from *Argania spinosa* forest soils, which have been reported as reservoirs of microbial biocontrol agents [37,48,49]. More generally, differences in recovered EPF profiles across sites can reflect both environmental context and methodological factors, including bait-insect choice [5,45]. In particular, insect-bait methods may preferentially recover EPF strains adapted to the selected host, which could contribute to differences in the relative abundance of *Beauveria* vs. *Metarhizium* observed among collections [50]. Moreover, this is the first time that *M. robertsii* and *M. guizhouense* were reported in Moroccan habitats (Figure S3), underscoring a hidden diversity that needs more investigation by screening and targeting more Moroccan forests and national parks coupled to next generation techniques (e.g., HTS and metagenomics) [51].

Given the urgent need for effective biocontrol agents against *T. absoluta* [24], we evaluated the pathogenicity and virulence of indigenous EPF isolates using a standardized leaf-based bioassay. Virulence varied widely across the collection, and several indigenous isolates performed comparably to, or better than, the reference strains (Figure 4; Table 1). Thus, to enable robust comparison across isolates, we ranked treatments using hazard ratios from Cox proportional hazards models relative to the reference strain *B. bassiana* ATCC 74040 and prioritized the top-performing candidates for dose–response testing (Table S6). Dose–response bioassays confirmed that the two lead isolates (*B. bassiana* UCA-350 and *M. robertsii* UCA-329) consistently ranked among the most potent candidates, with LC estimates comparable to, or exceeding, those of the benchmark reference strains (Table 1; Figure 5). Notably, performance differences among commercial reference strains also emphasize that virulence is strain-specific and should be validated under standardized conditions rather than inferred from species identity or commercial status.

Multiple research laboratories have been interested in the biocontrol of *T. absoluta* using entomopathogenic fungi [52,53,54]. Although extensive data are available on these newly identified strains, the parameters of these strains cannot be compared due to the lack of standardized methods. Most of these bioassays have used different methods (Spraying or dipping) and larval stages (L1 to L4) and have not included any reference or known EPF strain. Ndereyimana et al. [54] observed cumulative mortalities of 82%, 61%, and 47% by day 6 for *M. anisopliae* FCM Ar 23B3, *B. bassiana* J25, and *B. bassiana* GHA, respectively. In our study, the same cumulative mortality (50%) of *T. absoluta* was obtained at day 4 for GHA, whereas our best-performing strains, *B. bassiana* UCA-350 and *M. robertsii* UCA-329, showed higher values, with 100% and 90% cumulative mortality at day 4, respectively.

Notably, studies examining local EPF isolates have reported even lower LC_50_ values. Avery et al. [55] and Aynalem [56] found LC_50_ values ranging from 1.2 × 10^3^ to 7 × 10^4^ conidia/mL for *M. anisopliae* and *B. bassiana* isolates against Ethiopian populations of *T. absoluta*. While these values appeared to be lower than those reported in our study, they cannot be compared to the values in this study, due to different experimental conditions. Indeed, their bioassays used L2-L3 larvae and spraying with 3 mL of conidial suspension, whereas in this study, the L3-L4 larvae were exposed to leaflets that were dipped in conidial suspensions for 10 s.

Zheng et al. [57] evaluated the pathogenicity of three *Metarhizium* species, one *Beauveria* species, and one *Cordyceps* species against second instar *T. absoluta* larvae, reporting cumulative mortalities of 100% for *M. anisopliae*, 92.16% for both *M. flavoviride* and *Cordyceps fumosorosea*, and 92.62% for *B. bassiana* by day 7 using a 10^8^ conidia/mL concentration. These results are consistent with our findings, where 100% mortality was observed by day 6 for 16 tested isolates of our collection.

Other studies have explored the pathogenicity of local *B. bassiana* isolates from Brazil and Argentina against *T. absoluta*. Allegrucci et al. [58] and Silva et al. [59] reported median survival times ranging from 6 to 8 days when *B. bassiana* isolates were applied at a concentration of 1 × 10^8^ conidia/mL on leaflets infested with *T. absoluta* larvae. These MSTs are similar to those observed for most of our isolates. Notably, our most virulent isolates, UCA-350 and UCA-329, achieved better MSTs (2 to 3 days), underscoring genuine differences among isolates while also reflecting known variation arising from assay conditions, host populations, and strain identity.

Although virulence is a critical criterion for selecting effective entomopathogenic fungal strains, conidial production is equally important for successful field application and large-scale manufacturing [3,60]. In our study, the conidia production varied substantially among isolates (Figure 2; Tables S3 and S4), and the two lead candidates ranked among the higher-producing profiles. Notably, we observed a positive association between in vitro conidial yield and virulence (Table S7; Figure 6 and Figure S4), suggesting that production potential can align with pathogenic performance under standardized conditions. Together, these results place our two lead candidates among the higher-producing profiles, which supports the concept that strains capable of producing substantial quantities of viable conidia may possess a competitive advantage in both in vitro assays and pest infection dynamics [61]. Biologically, this association may reflect a broader propagule vigor axis, where isolates that sporulate abundantly also generate conidia with higher physiological quality (e.g., viability, energy reserves, and faster germination kinetics), which can translate into earlier host penetration and accelerated disease progression [8]. Importantly, production and virulence are often co-shaped by the same upstream factors. For instance, nutrient environment can influence growth, sporulation, germination speed, and virulence in EPF, indicating that the direction and strength of production–virulence relationships can be strain- and condition-dependent [62,63]. Nonetheless, plate-based yields should be further validated under mass production conditions using solid-state fermentation systems, as conidia production at the laboratory scale does not always translate directly to bioreactor performance [3].

Thermotolerance is also an important determinant of EPF field performance, as fungal conidia are frequently exposed to elevated daytime temperatures in agricultural environments [36,61,64]. In our study, four isolates (UCA-269, UCA-282, UCA-222 and UCA-252) showed no statistically significant reduction in germination after a 2 h exposure to 45 °C compared with their non-heated controls (Figure 3, Table S5), indicating a high level of heat tolerance. Fernandes et al. [65] evaluated the thermotolerance of 60 *Beauveria* isolates under the same temperature-time regime, found that many retained >60% germination, including the commercial strain *B. bassiana* GHA. Consistent with their results, we also identified *B. bassiana* UCA-317, *B. bassiana* UCA-333 and the reference strain *B. bassiana* GHA as thermotolerant, with post-stress germination exceeding this 60% threshold. It is noteworthy that heat stress may delay rather than completely prevent germination, as shown for *Metarhizium* spp. by Rangel et al. [40], suggesting that some isolates classified as thermosensitive under short incubation periods could partially recover over longer time frames. Importantly, the relationship between thermotolerance and virulence is expected to be context dependent. Thermotolerant conidia may survive longer on leaf surfaces and retain infectivity under hot microclimates, potentially improving infection opportunity under field conditions [66]. However, in the present study, virulence was quantified under a single bioassay temperature (26 ± 2 °C). Therefore, future work should test virulence across a range of temperatures and humidity regimes (including heat-stress scenarios) to determine whether thermotolerance predicts pathogenic performance under realistic field fluctuations [67,68].

Taken together, these findings underscore the need for a multidimensional approach in selecting entomopathogenic fungi. While virulence remains the primary filter for effective pest control, conidial productivity is equally crucial for ensuring sufficient inoculum and scalability, and thermotolerance further underpins performance under hot field conditions. Future work should explore the genetic and physiological determinants that enable certain isolates to perform well across these key traits, and assess whether formulation strategies such as biopolymer-based encapsulation of EPF [69] or alternative delivery approaches, including endophytic establishment [70], can improve the field-relevant performance of otherwise stress-sensitive strains. Moreover, greenhouse and field trials are underway to validate these laboratory results under real-world conditions.

## 5. Concluding Remarks and Trajectory for Future

Our integrated assessment reveals that the indigenous Moroccan isolates *Beauveria bassiana* UCA-350 and *Metarhizium robertsii* UCA-329 exhibit a compelling critical attribute: high virulence towards *Tuta absoluta*, vigorous conidial production, and moderate thermotolerance. This outcome underscores the profound value of targeted bioprospecting in under-explored ecologically distinct niches, such as the Argania forest, and validates a dual-isolation approach as a powerful strategy for capturing functional EPF diversity.

Translating these promising in vitro results into a reliable agricultural technology necessitates a coordinated research pathway. The immediate and paramount step is the empirical validation of efficacy through progressive greenhouse and open-field trials, confirming performance under real environmental and cropping conditions. Concurrently, this effort must be partnered with advanced formulation science to develop protective systems that enhance shelf-life and field resistance.

This holistic selection paradigm directly facilitates the development of effective, locally adapted mycoinsecticides, offering a sustainable and sophisticated tool for the integrated management of *Tuta absoluta* and other pervasive agricultural pests.

## Figures and Tables

**Table 1:** Effective concentrations for 50% and 90% mortality (LC_50_, LC_90_; conidia/mL) of *T. absoluta* larvae exposed to *Beauveria* and *Metarhizium* isolates, with standard errors (SE) and 95% confidence intervals (95% CI). Reference strains are marked with an asterisk (*).

Isolate	Species	LC_50_	LC_50_ SE	LC_50_ 95% CI	LC_90_	LC_90_ SE	LC_90_ 95% CI
UCA-350	*B. bassiana*	1.49 × 10^6^	3.54 × 10^5^	7.91 × 10^5^–2.18 × 10^6^	7.73 × 10^6^	3.08 × 10^6^	1.70 × 10^6^–1.38 × 10^7^
UCA-334	*B. bassiana*	2.91 × 10^6^	8.20 × 10^5^	1.30 × 10^6^–4.51 × 10^6^	2.69 × 10^7^	1.25 × 10^7^	2.33 × 10^6^–5.14 × 10^7^
UCA-333	*B. bassiana*	2.91 × 10^6^	7.91 × 10^5^	1.36 × 10^6^–4.46 × 10^6^	2.31 × 10^7^	1.02 × 10^7^	3.07 × 10^6^–4.31 × 10^7^
UCA-338	*B. bassiana*	3.54 × 10^6^	1.02 × 10^6^	1.55 × 10^6^–5.53 × 10^6^	3.52 × 10^7^	1.69 × 10^7^	2.12 × 10^6^–6.83 × 10^7^
GHA *	*B. bassiana*	4.06 × 10^6^	1.14 × 10^6^	1.83 × 10^6^–6.28 × 10^6^	3.67 × 10^7^	1.71 × 10^7^	3.30 × 10^6^–7.01 × 10^7^
UCA-346	*B. bassiana*	4.07 × 10^6^	1.10 × 10^6^	1.91 × 10^6^–6.22 × 10^6^	3.16 × 10^7^	1.39 × 10^7^	4.40 × 10^6^–5.88 × 10^7^
UCA-323	*B. bassiana*	4.08 × 10^6^	1.15 × 10^6^	1.83 × 10^6^–6.33 × 10^6^	3.70 × 10^7^	1.72 × 10^7^	3.21 × 10^6^–7.07 × 10^7^
UCA-316	*B. bassiana*	5.24 × 10^6^	1.47 × 10^6^	2.36 × 10^6^–8.11 × 10^6^	4.76 × 10^7^	2.24 × 10^7^	3.70 × 10^6^–9.15 × 10^7^
ATCC74040 *	*B. bassiana*	1.42 × 10^7^	3.89 × 10^6^	6.60 × 10^6^–2.18 × 10^7^	1.13 × 10^8^	5.46 × 10^7^	5.59 × 10^6^–2.20 × 10^8^
ARSEF4556 *	*M. brunneum*	2.25 × 10^6^	6.64 × 10^5^	9.53 × 10^5^–3.56 × 10^6^	2.52 × 10^7^	1.25 × 10^7^	7.56 × 10^5^–4.96 × 10^7^
UCA-329	*M. robertsii*	2.67 × 10^6^	7.12 × 10^5^	1.28 × 10^6^–4.07 × 10^6^	1.95 × 10^7^	8.37 × 10^6^	3.07 × 10^6^–3.59 × 10^7^
UCA-270	*M. anisopliae*	2.91 × 10^6^	7.62 × 10^5^	1.41 × 10^6^–4.40 × 10^6^	1.98 × 10^7^	8.28 × 10^6^	3.54 × 10^6^–3.60 × 10^7^
UCA-219	*M. anisopliae*	3.25 × 10^6^	8.85 × 10^5^	1.52 × 10^6^–4.99 × 10^6^	2.59 × 10^7^	1.15 × 10^7^	3.44 × 10^6^–4.84 × 10^7^
UCA-339	*M. anisopliae*	3.25 × 10^6^	9.18 × 10^5^	1.45 × 10^6^–5.05 × 10^6^	3.02 × 10^7^	1.41 × 10^7^	2.56 × 10^6^–5.77 × 10^7^
UCA-328	*M. anisopliae*	3.44 × 10^6^	9.35 × 10^5^	1.61 × 10^6^–5.27 × 10^6^	2.73 × 10^7^	1.21 × 10^7^	3.65 × 10^6^–5.10 × 10^7^
V275 *	*M. brunneum*	4.07 × 10^6^	1.18 × 10^6^	1.75 × 10^6^–6.39 × 10^6^	4.29 × 10^7^	2.11 × 10^7^	1.59 × 10^6^–8.42 × 10^7^
UCA-336	*M. guizhouense*	4.18 × 10^6^	1.15 × 10^6^	1.93 × 10^6^–6.44 × 10^6^	3.49 × 10^7^	1.57 × 10^7^	4.02 × 10^6^–6.57 × 10^7^
UCA-347	*M. guizhouense*	4.19 × 10^6^	1.21 × 10^6^	1.82 × 10^6^–6.55 × 10^6^	4.28 × 10^7^	2.08 × 10^7^	2.00 × 10^6^–8.36 × 10^7^
UCA-342	*M. anisopliae*	4.55 × 10^6^	1.22 × 10^6^	2.16 × 10^6^–6.94 × 10^6^	3.44 × 10^7^	1.50 × 10^7^	5.01 × 10^6^–6.37 × 10^7^
UCA-315	*M. guizhouense*	7.54 × 10^6^	2.14 × 10^6^	3.35 × 10^6^–1.17 × 10^7^	7.18 × 10^7^	3.52 × 10^7^	2.75 × 10^6^–1.41 × 10^8^

## Data Availability

The original contributions presented in this study are included in the article/Supplementary Materials. Further inquiries can be directed to the corresponding authors.

## Supplementary Materials

The following supporting information can be downloaded at https://www.mdpi.com/article/10.3390/jof12060452/s1, Figure S1: The entomopathogenic fungal isolates recovered from Moroccan soils before and after the preliminary pathogenicity screening on *T. molitor*. The colors indicate the method used to select the EPF (blue for the selective medium cultivation method; yellow for *T. molitor* baiting method); Figure S2: Macroscopic (A–D) and microscopic (a–d) morphological features of representative *Beauveria* and *Metarhizium* isolates. *Metarhizium* isolates form greenish colonies (A,B) with cylindrical-ellipsoidal conidia in chains produced on flask-shaped phialides (a,b). *Beauveria* colonies exhibit white to cream, powdery growth (C,D) with globose conidia produced sympodially on a zig-zag denticulate rachis, which extend from a globose-flask-shaped phialide (c,d); Figure S3: EF1-α (TEF1) phylogenetic tree of Moroccan *Metarhizium* isolates (primers EF1-983F/EF1-2218R), multiple sequence alignment performed with Clustal Omega v1.2.4; Figure S4: PCA visualization of conidia production, thermotolerance, and virulence (logHR) for local and reference EPF isolates; 95% ellipses denote performing strains vs. other isolates; Table S1: Overview of sampling locations (GPS coordinates, sampling date, and dominant crops) and recovery of entomopathogenic fungal isolates via selective medium and *T. molitor* baiting before and after preliminary screening across four regions in Morocco; Table S2: Preliminary pathogenicity screening results showing daily mortality (%) of *T. molitor* larvae over 10 days for each fungal isolate (only isolates achieving 100% mortality by Day 10 are presented) with metadata; Table S3: Estimated marginal means of conidia production by isolate (conidia per plate) with 95% confidence intervals and Tukey HSD compact-letter display (CLD), from one-way ANOVAs stratified by genus. Reference isolates are marked with an asterisk (*). Values and CIs are back-transformed from log10 models; Table S4: Tukey-adjusted pairwise comparisons for conidia production (log10(conidia per plate)) by isolate within each genus. Entries report the difference in estimated marginal means, standard error, degrees of freedom, t ratio, and Tukey-adjusted *p*-value; Table S5: Per-isolate germination in control vs. heat treatment with Wilson 95% CIs, and Benjamini–Hochberg (BH) adjusted Fisher’s exact *p*-values; Table S6: Cox proportional hazards analysis of *T. absoluta* larval survival (10-day assay) at 1 × 10^8^ conidia/mL for local isolates and reference strains. Hazard ratios (HR) with 95% CIs and *p*-values are reported for each isolate relative to *B. bassiana* ATCC 74040 (baseline, HR = 1); larval death was the event, and survivors at day 10 were right-censored. Reference strains are marked with an asterisk (*). Isolates with log(HR) ≥ 2 were preselected for dose–response testing, alongside the five reference strains (cells shaded with darker gray); Table S7: Pearson correlation matrix for log10 conidia production, log thermotolerance, and virulence (log Hazard Ratios): Pearson’s r, 95% CI (Fisher z), two-sided *p*-value.

## References

[1] Payumo J., Bello-Bravo J., Chennuru V., Mercene S.A., Yim C., Duynslager L., Kanamarlapudi B., Posos-Parra O., Payumo S., Mota-Sanchez D. (2024). An Assessment Model for Agricultural Databases: The Arthropod Pesticide Resistance Database as a Case Study. Insects.

[2] Sparks T.C., Crossthwaite A.J., Nauen R., Banba S., Cordova D., Earley F., Ebbinghaus-Kintscher U., Fujioka S., Hirao A., Karmon D. (2020). Insecticides, Biologics and Nematicides: Updates to IRAC’s Mode of Action Classification—A Tool for Resistance Management. Pestic. Biochem. Physiol..

[3] Lacey L.A., Grzywacz D., Shapiro-Ilan D.I., Frutos R., Brownbridge M., Goettel M.S. (2015). Insect Pathogens as Biological Control Agents: Back to the Future. J. Invertebr. Pathol..

[4] Mascarin G.M., Lopes R.B., Delalibera Í., Fernandes É.K.K., Luz C., Faria M. (2019). Current Status and Perspectives of Fungal Entomopathogens Used for Microbial Control of Arthropod Pests in Brazil. J. Invertebr. Pathol..

[5] Meyling N.V., Eilenberg J. (2006). Occurrence and Distribution of Soil Borne Entomopathogenic Fungi within a Single Organic Agroecosystem. Agric. Ecosyst. Environ..

[6] Scheepmaker J.W.A., Butt T.M. (2010). Natural and Released Inoculum Levels of Entomopathogenic Fungal Biocontrol Agents in Soil in Relation to Risk Assessment and in Accordance with EU Regulations. Biocontrol Sci. Technol..

[7] Zimmermann G. (2008). The Entomopathogenic Fungi *Isaria farinosa* (Formerly *Paecilomyces farinosus*) and the *Isaria fumosorosea* Species Complex (Formerly *Paecilomyces fumosoroseus*): Biology, Ecology and Use in Biological Control. Biocontrol Sci. Technol..

[8] Butt T.M., Coates C.J., Dubovskiy I.M., Ratcliffe N.A., Lovett B., St. Leger R. (2016). Entomopathogenic Fungi: New Insights into Host-Pathogen Interactions. Advances in Genetics.

[9] Ma M., Luo J., Li C., Eleftherianos I., Zhang W., Xu L. (2024). A Life-and-Death Struggle: Interaction of Insects with Entomopathogenic Fungi across Various Infection Stages. Front. Immunol..

[10] Ortiz-Urquiza A., Keyhani N. (2013). Action on the Surface: Entomopathogenic Fungi versus the Insect Cuticle. Insects.

[11] Pedrini N. (2018). Molecular Interactions between Entomopathogenic Fungi (Hypocreales) and Their Insect Host: Perspectives from Stressful Cuticle and Hemolymph Battlefields and the Potential of Dual RNA Sequencing for Future Studies. Fungal Biol..

[12] Dubovskiy I.M., Butt T. (2023). Entomopathogenic Fungi in Biological Plant Protection: The Machinery of Multicomponent System Interactions. J. Fungi.

[13] Hong S., Shang J., Sun Y., Tang G., Wang C. (2024). Fungal Infection of Insects: Molecular Insights and Prospects. Trends Microbiol..

[14] Wu S., Toews M.D., Oliveira-Hofman C., Behle R.W., Simmons A.M., Shapiro-Ilan D.I. (2020). Environmental Tolerance of Entomopathogenic Fungi: A New Strain of Cordyceps Javanica Isolated from a Whitefly Epizootic Versus Commercial Fungal Strains. Insects.

[15] Faria M.R.D., Wraight S.P. (2007). Mycoinsecticides and Mycoacaricides: A Comprehensive List with Worldwide Coverage and International Classification of Formulation Types. Biol. Control.

[16] Chang J.-C., Wu S.-S., Liu Y.-C., Yang Y.-H., Tsai Y.-F., Li Y.-H., Tseng C.-T., Tang L.-C., Nai Y.-S. (2021). Construction and Selection of an Entomopathogenic Fungal Library from Soil Samples for Controlling Spodoptera Litura. Front. Sustain. Food Syst..

[17] Kim J.C., Lee M.R., Kim S., Lee S.J., Park S.E., Nai Y.-S., Lee G.S., Shin T.Y., Kim J.S. (2018). Tenebrio Molitor-Mediated Entomopathogenic Fungal Library Construction for Pest Management. J. Asia-Pac. Entomol..

[18] Li Z., Alves S.B., Roberts D.W., Fan M., Delalibera I., Tang J., Lopes R.B., Faria M., Rangel D.E.N. (2010). Biological Control of Insects in Brazil and China: History, Current Programs and Reasons for Their Successes Using Entomopathogenic Fungi. Biocontrol Sci. Technol..

[19] Quesada-Moraga E., González-Mas N., Yousef-Yousef M., Garrido-Jurado I., Fernández-Bravo M. (2024). Key Role of Environmental Competence in Successful Use of Entomopathogenic Fungi in Microbial Pest Control. J. Pest Sci..

[20] Bayman P., Mariño Y.A., García-Rodríguez N.M., Oduardo-Sierra O.F., Rehner S.A. (2021). Local Isolates of Beauveria Bassiana for Control of the Coffee Berry Borer Hypothenemus Hampei in Puerto Rico: Virulence, Efficacy and Persistence. Biol. Control.

[21] Clifton E.H., Castrillo L.A., Jaronski S.T., Hajek A.E. (2023). Cryptic Diversity and Virulence of Beauveria Bassiana Recovered from Lycorma Delicatula (Spotted Lanternfly) in Eastern Pennsylvania. Front. Insect Sci..

[22] Quesada-Moraga E., Maranhao E.A.A., Valverde-García P., Santiago-Álvarez C. (2006). Selection of Beauveria Bassiana Isolates for Control of the Whiteflies Bemisia Tabaci and Trialeurodes Vaporariorum on the Basis of Their Virulence, Thermal Requirements, and Toxicogenic Activity. Biol. Control.

[23] Ranesi M., Vitale S., Staropoli A., Di Lelio I., Izzo L.G., De Luca M.G., Becchimanzi A., Pennacchio F., Lorito M., Woo S.L. (2024). Field Isolates of Beauveria Bassiana Exhibit Biological Heterogeneity in Multitrophic Interactions of Agricultural Importance. Microbiol. Res..

[24] Biondi A., Guedes R.N.C., Wan F.-H., Desneux N. (2018). Ecology, Worldwide Spread, and Management of the Invasive South American Tomato Pinworm, *Tuta absoluta*: Past, Present, and Future. Annu. Rev. Entomol..

[25] Desneux N., Wajnberg E., Wyckhuys K.A.G., Burgio G., Arpaia S., Narváez-Vasquez C.A., González-Cabrera J., Catalán Ruescas D., Tabone E., Frandon J. (2010). Biological Invasion of European Tomato Crops by Tuta Absoluta: Ecology, Geographic Expansion and Prospects for Biological Control. J. Pest Sci..

[26] Mansour R., Brévault T., Chailleux A., Cherif A., Grissa-Lebdi K., Haddi K., Mohamed S.A., Nofemela R.S., Oke A., Sylla S. (2018). Occurrence, Biology, Natural Enemies and Management of Tuta Absoluta in Africa. Entomol. Gen..

[27] Martins J.C., Picanço M.C., Bacci L., Guedes R.N.C., Santana P.A., Ferreira D.O., Chediak M. (2016). Life Table Determination of Thermal Requirements of the Tomato Borer Tuta Absoluta. J. Pest Sci..

[28] Mohamed S.A., Azrag A.G.A., Obala F., Ndlela S. (2022). Estimating the Demographic Parameters of Tuta Absoluta (Lepidoptera: Gelechiidae) Using Temperature-Dependent Development Models and Their Validation under Fluctuating Temperature. Biology.

[29] Tarusikirwa V.L., Machekano H., Mutamiswa R., Chidawanyika F., Nyamukondiwa C. (2020). Tuta Absoluta (Meyrick) (Lepidoptera: Gelechiidae) on the “Offensive” in Africa: Prospects for Integrated Management Initiatives. Insects.

[30] Faria M., Lopes R.B., Souza D.A., Wraight S.P. (2015). Conidial Vigor vs. Viability as Predictors of Virulence of Entomopathogenic Fungi. J. Invertebr. Pathol..

[31] Mascarin G.M., Golo P.S., De Souza Ribeiro-Silva C., Muniz E.R., De Oliveira Franco A., Kobori N.N., Fernandes É.K.K. (2024). Advances in Submerged Liquid Fermentation and Formulation of Entomopathogenic Fungi. Appl. Microbiol. Biotechnol..

[32] Jordan C., Dos Santos P.L., Oliveira L.R.D.S., Domingues M.M., Gêa B.C.C., Ribeiro M.F., Mascarin G.M., Wilcken C.F. (2021). Entomopathogenic Fungi as the Microbial Frontline against the Alien Eucalyptus Pest Gonipterus Platensis in Brazil. Sci. Rep..

[33] Ramos Y., Pineda-Guillermo S., Tamez-Guerra P., Orozco-Flores A.A., Figueroa De La Rosa J.I., Ramos-Ortiz S., Chavarrieta-Yáñez J.M., Martínez-Castillo A.M. (2024). Natural Prevalence, Molecular Characteristics, and Biological Activity of *Metarhizium rileyi* (Farlow) Isolated from *Spodoptera frugiperda* (J. E. Smith) Larvae in Mexico. J. Fungi.

[34] Wu S., Toews M.D., Behle R.W., Barman A.K., Sparks A.N., Simmons A.M., Shapiro-Ilan D.I. (2023). Post-Application Field Persistence and Efficacy of *Cordyceps javanica* against *Bemisia tabaci*. J. Fungi.

[35] Acheampong M.A., Coombes C.A., Moore S.D., Hill M.P. (2020). Temperature Tolerance and Humidity Requirements of Select Entomopathogenic Fungal Isolates for Future Use in Citrus IPM Programmes. J. Invertebr. Pathol..

[36] Inglis G.D., Enkerli J., Goettel M.S. (2012). Laboratory Techniques Used for Entomopathogenic Fungi: Hypocreales.

[37] Imoulan A., Alaoui A., El Meziane A. (2011). Natural Occurrence of Soil-Borne Entomopathogenic Fungi in the Moroccan Endemic Forest of Argania Spinosa and Their Pathogenicity to Ceratitis Capitata. World J. Microbiol. Biotechnol..

[38] Ayala-Zermeño M.A., Gallou A., Berlanga-Padilla A.M., Andrade-Michel G.Y., Rodríguez-Rodríguez J.C., Arredondo-Bernal H.C., Montesinos-Matías R. (2017). Viability, Purity, and Genetic Stability of Entomopathogenic Fungi Species Using Different Preservation Methods. Fungal Biol..

[39] Oliveira D.G.P., Pauli G., Mascarin G.M., Delalibera I. (2015). A Protocol for Determination of Conidial Viability of the Fungal Entomopathogens Beauveria Bassiana and Metarhizium Anisopliae from Commercial Products. J. Microbiol. Methods.

[40] Rangel D.E.N., Braga G.U.L., Anderson A.J., Roberts D.W. (2005). Variability in Conidial Thermotolerance of Metarhizium Anisopliae Isolates from Different Geographic Origins. J. Invertebr. Pathol..

[41] Insecticide Resistance Action Committee (IRAC) (2012). IRAC Susceptibility Test Methods Series: Method No. 022—Tuta Absoluta Larvae (Leaf-Dip Bioassay).

[42] Humber R.A., Lacey L.A. (2012). Identification of entomopathogenic fungi. Manual of Techniques in Invertebrate Pathology.

[43] R Core Team (2025). R: A Language and Environment for Statistical Computing.

[44] Zimmermann G. (2007). Review on safety of the entomopathogenic fungi *Beauveria bassiana* and *Beauveria brongniartii*. Biocontrol Sci. Technol..

[45] Quesada-Moraga E., Navas-Cortés J.A., Maranhao E.A.A., Ortiz-Urquiza A., Santiago-Álvarez C. (2007). Factors Affecting the Occurrence and Distribution of Entomopathogenic Fungi in Natural and Cultivated Soils. Mycol. Res..

[46] Shin T.-Y., Choi J.-B., Bae S.-M., Koo H.-N., Woo S.-D. (2010). Study on Selective Media for Isolation of Entomopathogenic Fungi. Int. J. Ind. Entomol..

[47] Masoudi A., Koprowski J.L., Bhattarai U.R., Wang D. (2018). Elevational Distribution and Morphological Attributes of the Entomopathogenic Fungi from Forests of the Qinling Mountains in China. Appl. Microbiol. Biotechnol..

[48] Aboussaid H., Vidal-Quist J.C., Oufdou K., El Messoussi S., Castañera P., González-Cabrera J. (2011). Occurrence, Characterization and Insecticidal Activity of *Bacillus thuringiensis* Strains Isolated from Argan Fields in Morocco. Environ. Technol..

[49] Imoulan A., Elmeziane A. (2014). Pathogenicity of Beauveria Bassiana Isolated from Moroccan Argan Forests Soil against Larvae of Ceratitis Capitata (Diptera: Tephritidae) in Laboratory Conditions. World J. Microbiol. Biotechnol..

[50] Sharma L., Oliveira I., Torres L., Marques G. (2018). Entomopathogenic Fungi in Portuguese Vineyards Soils: Suggesting a ‘*Galleria*-*Tenebrio*-Bait Method’ as Bait-Insects *Galleria* and *Tenebrio* Significantly Underestimate the Respective Recoveries of *Metarhizium* (*robertsii*) and *Beauveria* (*bassiana*). MycoKeys.

[51] Masoudi A., Wang M., Zhang X., Wang C., Qiu Z., Wang W., Wang H., Liu J. (2020). Meta-Analysis and Evaluation by Insect-Mediated Baiting Reveal Different Patterns of Hypocrealean Entomopathogenic Fungi in the Soils from Two Regions of China. Front. Microbiol..

[52] Akutse K.S., Subramanian S., Khamis F.M., Ekesi S., Mohamed S.A. (2020). Entomopathogenic Fungus Isolates for Adult *Tuta absoluta* (Lepidoptera: Gelechiidae) Management and Their Compatibility with *Tuta* Pheromone. J. Appl. Entomol..

[53] Kinyanjui G., Tadesse Mawcha K., Ndolo D. (2025). Advancing Entomopathogenic Fungi for Improved Management of *Phthorimaea (Tuta) absoluta* (Lepidoptera: Gelechiidae). J. Integr. Pest Manag..

[54] Ndereyimana A., Nyalala S., Murerwa P., Gaidashova S. (2019). Pathogenicity of Some Commercial Formulations of Entomopathogenic Fungi on the Tomato Leaf Miner, Tuta Absoluta (Meyrick) (Lepidoptera: Gelechiidae). Egypt. J. Biol. Pest Control.

[55] Avery P.B., Duren E.B., Qureshi J.A., Adair R.C., Adair M.M., Cave R.D. (2021). Field Efficacy of Cordyceps Javanica, White Oil and Spinetoram for the Management of the Asian Citrus Psyllid, *Diaphorina citri*. Insects.

[56] Aynalem B. (2022). Empirical Review of Tuta Absoluta Meyrick Effect on the Tomato Production and Their Protection Attempts. Adv. Agric..

[57] Zheng Y., Liu Y., Zhang J., Liu X., Ju Z., Shi H., Mendoza-Mendoza A., Zhou W. (2023). Dual Role of Endophytic Entomopathogenic Fungi: Induce Plant Growth and Control Tomato Leafminer *Phthorimaea absoluta*. Pest Manag. Sci..

[58] Allegrucci N., Velazquez M.S., Russo M.L., Perez E., Scorsetti A.C. (2017). Endophytic colonisation of tomato by the entomopathogenic fungus *Beauveria bassiana*: The use of different inoculation techniques and their effects on the tomato leafminer *Tuta absoluta* (Lepidoptera: Gelechiidae). J. Plant Prot. Res..

[59] Silva G.A., Queiroz E.A., Arcanjo L.P., Lopes M.C., Araújo T.A., Galdino T.S.V., Samuels R.I., Rodrigues-Silva N., Picanço M.C. (2021). Biological Performance and Oviposition Preference of Tomato Pinworm Tuta Absoluta When Offered a Range of Solanaceous Host Plants. Sci. Rep..

[60] Mantzoukas S., Lagogiannis I., Mpousia D., Ntoukas A., Karmakolia K., Eliopoulos P.A., Poulas K. (2021). Beauveria Bassiana Endophytic Strain as Plant Growth Promoter: The Case of the Grape Vine Vitis Vinifera. J. Fungi.

[61] Jaronski S.T., Morales-Ramos J.A., Rojas M.G., Shapiro-Ilan D.I. (2022). Mass Production of Entomopathogenic Fungi—State of the Art. Mass Production of Beneficial Organisms: Invertebrates and Entomopathogens.

[62] Safavi S.A., Shah F.A., Pakdel A.K., Reza Rasoulian G., Bandani A.R., Butt T.M. (2007). Effect of Nutrition on Growth and Virulence of the Entomopathogenic Fungus *Beauveria bassiana*. FEMS Microbiol. Lett..

[63] Safavi S.A. (2012). Attenuation of the Entomopathogenic Fungus *Beauveria bassiana* Following Serial in Vitro Transfers. Biologia.

[64] Da Paixão F.R.S., Muniz E.R., Catão A.M.L., Santos T.R., Luz C., Marreto R.N., Mascarin G.M., Fernandes É.K.K. (2023). Microsclerotial Pellets of *Metarhizium* spp.: Thermotolerance and Bioefficacy against the Cattle Tick. Appl. Microbiol. Biotechnol..

[65] Fernandes É.K.K., Rangel D.E.N., Moraes Á.M.L., Bittencourt V.R.E.P., Roberts D.W. (2008). Cold Activity of Beauveria and Metarhizium, and Thermotolerance of Beauveria. J. Invertebr. Pathol..

[66] Bidochka M.J., Menzies F.V., Kamp A.M. (2002). Genetic groups of the insect pathogenic fungus *Beauveria bassiana* are associated with habitat and thermal growth preferences. Arch. Microbiol..

[67] Kryukov V., Yaroslavtseva O., Tyurin M., Akhanaev Y., Elisaphenko E., Wen T.-C., Tomilova O., Tokarev Y., Glupov V. (2017). Ecological preferences of *Metarhizium* spp. from Russia and neighboring territories and their activity against Colorado potato beetle larvae. J. Invertebr. Pathol..

[68] Keyser C.A., Fernandes É.K.K., Rangel D.E.N., Roberts D.W. (2014). Heat-Induced Post-Stress Growth Delay: A Biological Trait of Many Metarhizium Isolates Reducing Biocontrol Efficacy?. J. Invertebr. Pathol..

[69] Kamouni A., Idali N., Brik A., Qayouh H., Dihazi A., Vinceković M., Youcef H.B., Meziane A.E., Lahcini M. (2025). Encapsulation of Beauveria Bassiana Conidia in Carboxymethylcellulose Beads: Advancing UV Protection and Thermal Resilience in Bioinsecticide Applications. Int. J. Biol. Macromol..

[70] Ghaffari S., Karimi J., Cheniany M., Seifi A., Loverodge J., Butt T.M. (2025). Endophytic Entomopathogenic Fungi Enhance Plant Immune Responses against Tomato Leafminer. J. Invertebr. Pathol..

