# Supplementary material for: Unlocking the Biocontrol Potential of Indigenous Soil Fungi: High-Performing Strains of Beauveria bassiana and Metarhizium robertsii Against the Tomato Leafminer Tuta absoluta
Source: J Fungi (Basel). 2026 Jun 21;12(6):452. doi: 10.3390/jof12060452 (PMC13301989; doi:10.3390/jof12060452)
Supplement: Supplementary file 1 [file jof-12-00452-s001.zip › Supplementary_Figures.pdf]

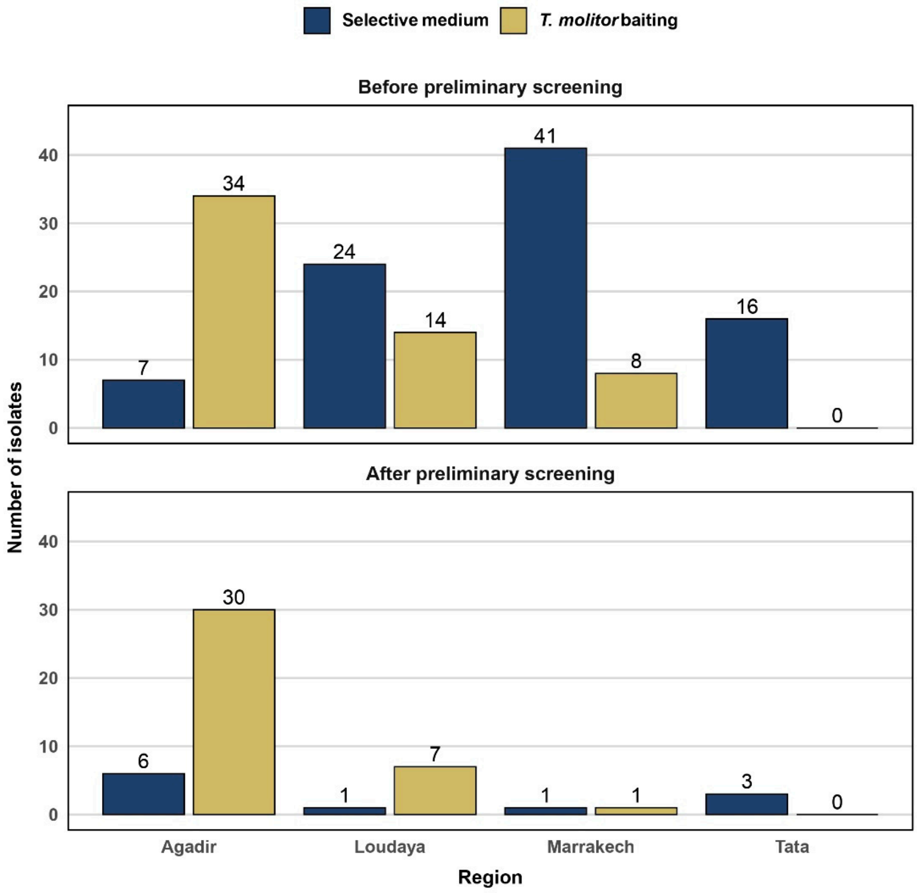

Figure S1: The entomopathogenic fungal isolates recovered from Moroccan soils before and after the preliminary pathogenicity screening on *T. molitor*. The colors indicate the method used to select the EPF (blue for selective medium cultivation method; yellow for *T. molitor* baiting method)

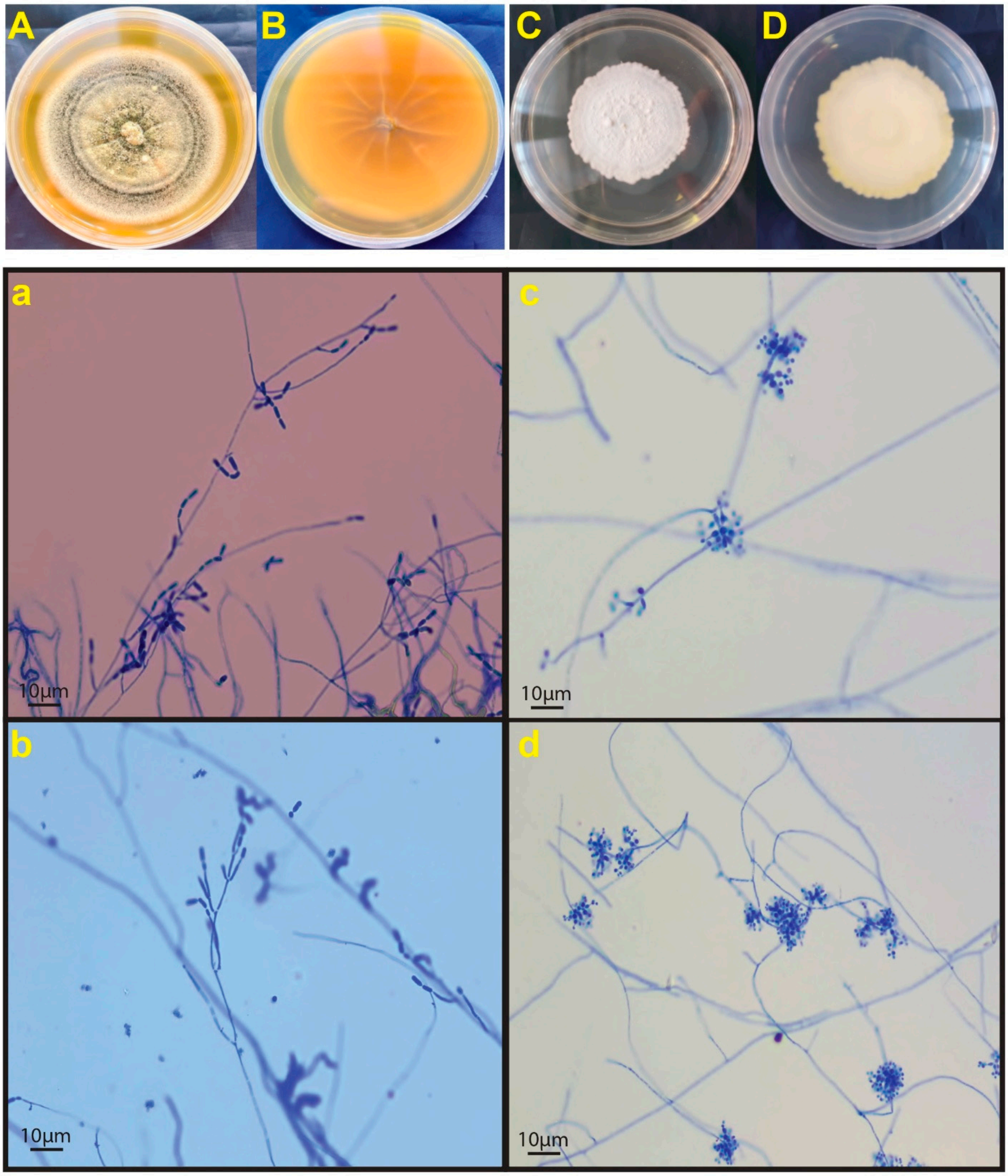

Figure S2: Macroscopic (A, B, C, D) and microscopic (a, b, c, d) morphological features of representative Beauveria and Metarhizium isolates. Metarhizium isolates form greenish colonies (A, B) with cylindrical-ellipsoidal conidia in chains produced on flask-shaped phialides (a, b). Beauveria colonies exhibit white to cream, powdery growth (C, D) with globose conidia produced sympodially on a zig-zag denticulate rachis, which extend from a globose-flask-shaped phialide (c, d)

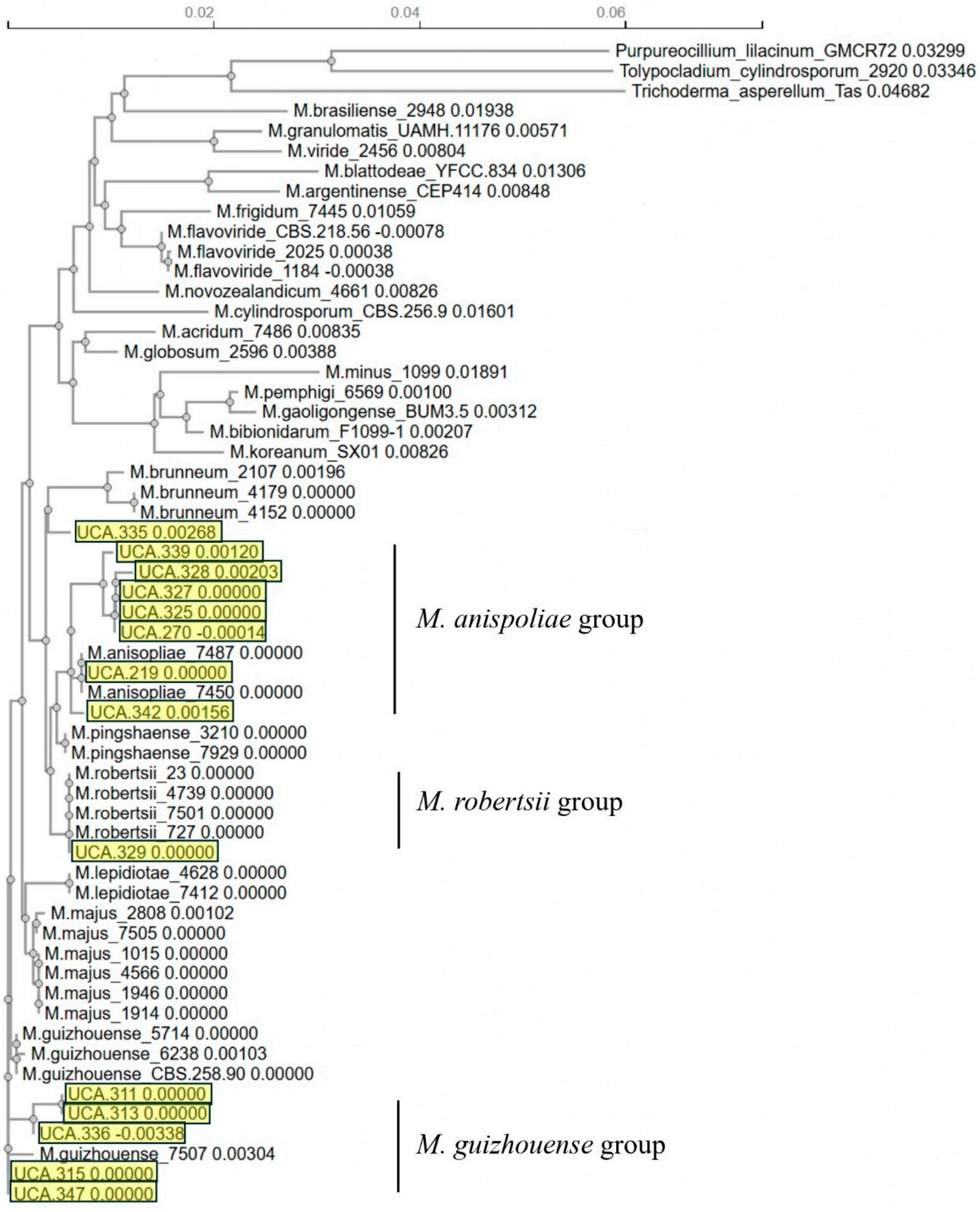

Figure S3: EF1-α (TEF1) phylogenetic tree of Moroccan *Metarhizium* isolates (primers EF1-983F / EF1-2218R), multiple sequence alignment performed with Clustal Omega.

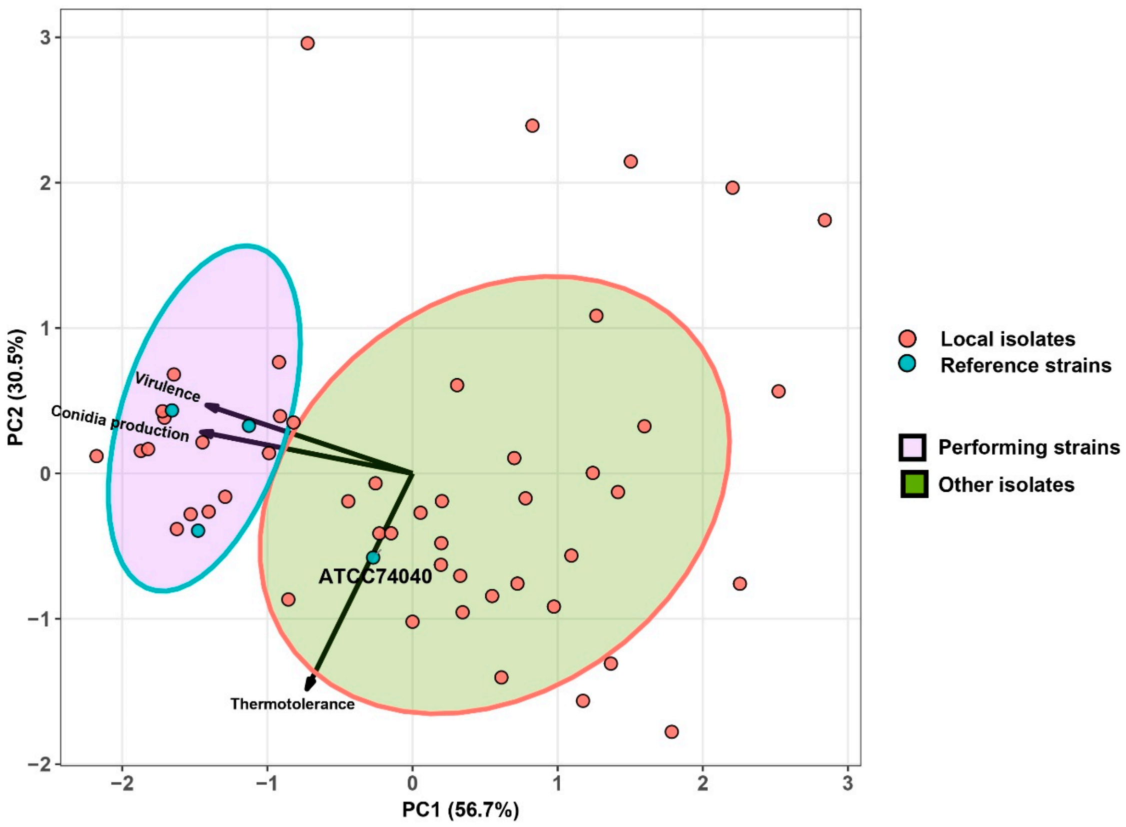

Figure S4: PCA visualization of conidia production, thermotolerance, and virulence (logHR) for local and reference EPF isolates; 95% ellipses denote performing strains vs other isolates.
